# The Hospital Section at the Woman's Exhibition

**Published:** 1900-06-02

**Authors:** 


### THE HOSPITAL SECTION AT THE WOMAN'S EXHIBITION.

The Charing Cross Hospital Special Appeal Committee are to be congratulated on the manner in which they have organised the section devoted to hospital work at the Earl's Court Exhibition. The corner in the "Ideal City" where the "Model Hospital" is to be found is already attracting many visitors, although at the moment of writing it is hardly yet in complete working order. The idea has been, in view of the keen interest taken by one and all just now in field hospitals, and all that appertains to the care of the sick and wounded on active service, to exhibit both civil and military methods, as it were, in combination. The treatment of the soldier is therefore shown from the moment he comes under medical observation to his final discharge, once more restored to health and activity.

Ambulance cart, field operating tent, dressing stations, &c., with all their expedients for prompt and rapid "First Aid," are exhibited outside the picturesque building which represents Charing Cross Hospital.

In the ward the eight beds are occupied by lay figures representing cases of wounds and injuries, and around each of them are displayed the most modern paraphernalia for their respective surgical necessities. Fractured skulls, thighs, ribs, amputation, abdominal injury, &c., afford opportunities for showing the special appliances for the different kinds of injury. The construction of the ward itself, with polished oak floor, set in antiseptic bituminous compound, rounded corners, windows guiltless of dust-collecting ledges, its system of ventilation, light reflecting walls, and aseptic furniture and fittings, is worth study, and full of much that is interesting. A completely equipped operating-room, electrically heated and ventilated, and with tables, instrument cases, &c., of glass and metal, comes next, with an anæsthetic room opening from it. Beyond is a complete installation of Röntgen ray apparatus, in working order, as now in use in many of our hospitals.

The convalescent is not forgotten, a typical day and bed room of a convalescent home being shown. In the bedroom is the small but interesting exhibit of "Appliances designed by Nurses," shown by the Editors of *Nursing Notes* of which a notice has already appeared in these columns.

Another very essential part of the model hospital is the "Sanitary Annex," demonstrating what bathrooms and lavatories should be, floored with terrazzo, and walled with tiles, all surfaces as washable as modern invention can make them, and with slop sinks, &c., of the latest patterns. Here also is the laundry, where much will be found to note and admire. Nor should the "Duty Room," on the right of the entrance, be left unnoticed, with its specimens of electrically-heated foot-warmers, kettles, and other requisites, and the arrangements for the disposal of patients' clothes, the linen cupboards, milk sterilisers, and refrigerators on the opposite side. The space at disposal not being very great, those to whose hands the organisation of this section has been entrusted have certainly made the most of their opportunities, with a result which should secure to Charing Cross Hospital that support and interest which it so eminently deserves. Mr. R. G. Lund, secretary of the Appeal Committee, has worked hard to ensure the success of this section, and the members of the staff of the hospital have personally taken much interest in the preparations. Miss Gordon has superintended the nursing details, and the ward is presided over by a Sister, with the help of several nurses. A full description of the entire section, illustrated with photographs of the nursing staff of the hospital, is issued as a souvenir in aid of the Special Appeal Fund.

